# Indole-3-Acetic Acid Is Synthesized by the Endophyte *Cyanodermella asteris* via a Tryptophan-Dependent and -Independent Way and Mediates the Interaction with a Non-Host Plant

**DOI:** 10.3390/ijms22052651

**Published:** 2021-03-06

**Authors:** Linda Jahn, Uta Hofmann, Jutta Ludwig-Müller

**Affiliations:** Institute of Botany, Faculty of Biology, Technische Universität Dresden, 01062 Dresden, Germany; Linda.Jahn@tu-dresden.de (L.J.); hofmann.uta@gmx.net (U.H.)

**Keywords:** *Arabidopsis thaliana*, *Cyanodermella asteris*, endophyte, IAA biosynthesis, IAA uptake, Trp-dependent pathway, Trp-independent pathway

## Abstract

The plant hormone indole-3-acetic acid (IAA) is one of the main signals playing a role in the communication between host and endophytes. Endophytes can synthesize IAA de novo to influence the IAA homeostasis in plants. Although much is known about IAA biosynthesis in microorganisms, there is still less known about the pathway by which IAA is synthesized in fungal endophytes. The aim of this study is to examine a possible IAA biosynthesis pathway in *Cyanodermella asteris*. In vitro cultures of *C. asteris* were incubated with the IAA precursors tryptophan (Trp) and indole, as well as possible intermediates, and they were additionally treated with IAA biosynthesis inhibitors (2-mercaptobenzimidazole and yucasin DF) to elucidate possible IAA biosynthesis pathways. It was shown that (a) *C. asteris* synthesized IAA without adding precursors; (b) indole-3-acetonitrile (IAN), indole-3-acetamide (IAM), and indole-3-acetaldehyde (IAD) increased IAA biosynthesis; and (c) *C. asteris* synthesized IAA also by a Trp-independent pathway. Together with the genome information of *C. asteris*, the possible IAA biosynthesis pathways found can improve the understanding of IAA biosynthesis in fungal endophytes. The uptake of fungal IAA into *Arabidopsis thaliana* is necessary for the induction of lateral roots and other fungus-related growth phenotypes, since the application of the influx inhibitor 2-naphthoxyacetic acid (NOA) but not the efflux inhibitor N-1-naphtylphthalamic acid (NPA) were altering these parameters. In addition, the root phenotype of the mutation in an influx carrier, *aux1*, was partially rescued by *C. asteris*.

## 1. Introduction

The plant hormone indole-3-acetic acid (IAA) plays a role in the communication between host plant and microbes, including plant-associated microorganisms [1,2,3] and endophytes [3,4], but also plant pathogens [5,6,7]. IAA together with other phytohormones is responsible for plant growth and development. It is involved in nearly each plant process such as cell growth, root initiation, tropism, fruit ripening, or senescence [8]. If plant-associated microorganism can regulate the plant IAA levels, they are able to influence the plant metabolism to their benefit, either as pathogens or as beneficial microorganisms. Then, microbial IAA acts as a mediator between plants and microorganisms and plays a key role in the communication between both partners. Microorganisms alters plant auxin levels and can therefore influence the plant development.

The concentration of IAA in plants can be controlled via different mechanisms, mainly biosynthesis, transport, conjugation, and degradation [9]. In addition, the auxin level of plants can be influenced by the de novo biosynthesis of IAA by microorganisms. However, for IAA produced by microbes, they either need to be residing within a host plant or the IAA needs to be taken up usually through the roots. The influx of IAA is possible either by diffusion via the plasma membrane and/or by uptake carrier proteins. The auxin resistant1/like auxin resistant (AUX/LAX) family mediates these in *Arabidopsis thaliana* [10]. Auxin carriers might be involved in the interaction between plants and fungi; for example, a LAX family member (LAX3) was differentially regulated in *A. thaliana* roots inoculated with the growth-promoting fungus *Serendipita indica* [11], and *Trichoderma virens* needs IAA influx carriers to mediate its interaction with *A. thaliana* [1].

**Figure 1:**
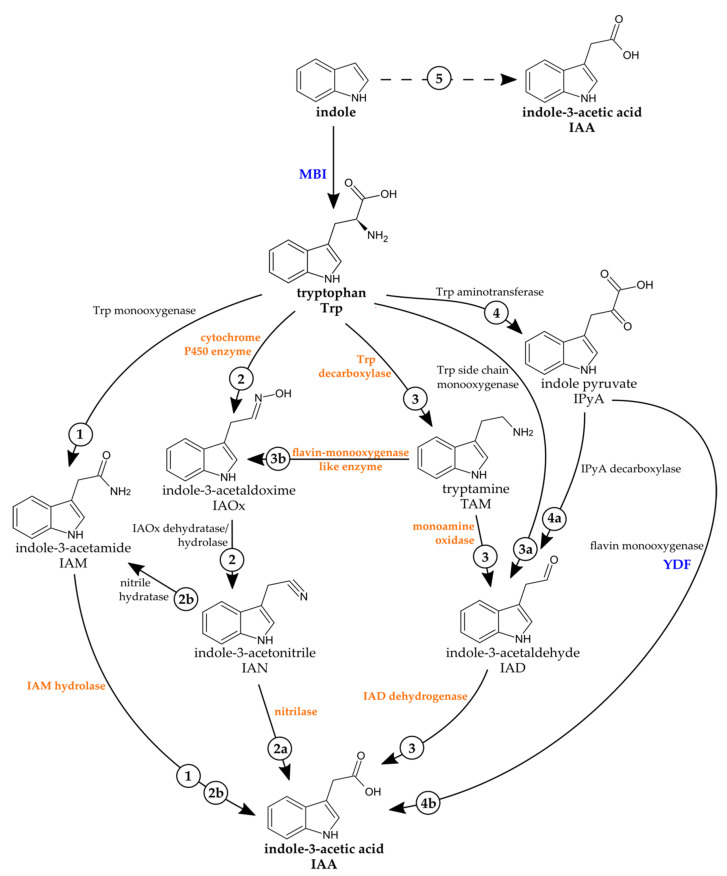
Indole-3-acetic acid (IAA) biosynthesis pathways in bacteria, fungi, and plants with the known enzymes (modified after KEGG [36,37,38]). IAA can be synthesized from tryptophan (Trp-dependent pathways, 1–4) and directly from indole (Trp-independent pathways, 5). Plants synthesize IAA over the indole-3-acetamide (IAM) (1), the indole-3-acetaldoxime/indole-3-acetonitrile (IAOx–IAN) (2-2a), the tryptamine (TAM) (3-3b-2-2a), and the indole-3-pyruvic acid (IPyA) pathway (4-4a-3 or 4-4b). Bacteria and fungi prefer the IAM (1), the IAOx–IAN (2-2a or 2-2b), the TAM (3), or the IPyA (4-4a-3) pathway. The IAA pathway with the direct conversion of Trp to indole-3-acetaldehyde (IAD, without IPyA as intermediate, 3a-3) is only known in *Pseudomonas* species [39,40,41,42]. The reaction site of the IAA biosynthesis inhibitors 2-mercaptobenzimidazole (MBI) (Trp synthase) and yucasin DF (YDF) (YUC enzymes) is shown in blue. Enzymes for which candidate genes were found in *C. asteris* are highlighted in orange (see also Table 1).

There are several well-known pathways to synthesize IAA in plants (reviewed in [12]). IAA can be synthesized by a tryptophan (Trp)-dependent and/or -independent pathway. The Trp-dependent pathway includes several different possible routes, named after the main intermediate: indole-3-pyruvic acid (IPyA), indole-3-acetamide (IAM), tryptamine (TAM), and indole-3-acetaldoxime/indole-3-acetonitrile (IAOx–IAN) pathways that may be interconnected (Figure 1). In several pathways, indole-3-acetaldehyde (IAD) is an intermediate. In the majority of plants, the major pathway is assumed to function via IPyA [13], but due to the existence of IAM [14] and also the IAOx-IAN pathway that is connected to indole glucosinolates in *A. thaliana*, the situation seems to be more complicated [15]. In the Trp-independent pathways, indole is most likely used as an IAA precursor [16,17,18]. Bacteria and fungi are also able to synthesize IAA [19], most likely using similar ways as found in plants, but there is not as much known. The genes and enzymes of the IAA biosynthesis steps are not completely resolved [20,21]. Fungi interacting with plants can produce IAA to increase root growth as endophytes in the rhizosphere or in the host plant itself. Pathogenic fungi can use IAA to cause disease symptoms or to interfere with the plant defense system [22]. Therefore, the focus was often on plant pathogens, especially for fungi [23]. For example, the smut fungus *Ustilago maydis* is causing galls on host tissues and can synthesize its own IAA [24]. Genes encoding proteins to synthesize IAA via the IAD pathway but also via TAM have been identified. Double knockout mutants had lower IAA levels, indicating the role of this pathway for fungal IAA production. Similarly, *Ustilago esculenta* converted IAD to IAA, but in addition IPyA and indole-3-lactic acid [25]. An early study on different *Taphrina* species, which also cause hyperplastic diseases in plants, indicated a pathway via IPyA and IAD, but the fungi could also convert IAN to IAA [26]. In four different plant-associated *Fusarium* species, among them corn, rice, and tomato pathogens, genes for the IAM pathway were identified [27]. *F. graminearum* could use TAM and IAN as intermediates rather than IAM [28]. *Colletotrichum gloeosporioides* causing anthracnose disease synthesized IAA in culture through IAM [29], similar to the rust fungus *Puccinia graminis* from which the respective genes were identified [30]. *Sclerotinia sclerotiorum* seemed to use the IAOx–IAN pathway due to an enzymatic activity for an IAOx dehydratase that was isolated and characterized [31].

The mechanisms, especially how endophytes synthesize IAA, are important to understand the communication between them and their host [32,33]. However, for endophytic fungi, not so many studies have been performed. *T. virens* was capable of interacting with *A. thaliana* by its own IAA synthesis [1]. Since the fungus produced IAD and indole-3-ethanol in addition to IAA, it can be assumed that at least the IAD pathway is active in this endophyte. The arbuscular mycorrhizal fungus *Rhizophagus irregularis* was analyzed for the presence of IAA production, but no biosynthetic pathway was elucidated so far [34]. In the beneficial endophytic basidiomycete *S. indica*, IAA production was shown also, again without describing a specific pathway [35].

*Cyanodermella asteris* is an endophyte from the inflorescence axis of *Aster tataricus* [43], which is a perennial plant from the Northern part of Asia. Roots and rhizomes of *A. tataricus* are well known in the traditional Chinese medicine due to their antibacterial, antiviral, antiulcer, and diuretic activities [44,45,46]. The antitumor compounds astins, originally found in *A. tataricus* [47,48], can be synthesized by the endophytic fungus *C. asteris*, but some bioactive variants are only found in the plant [49]. Therefore, understanding the communication between both partners could shed light on possible mechanisms of how the synthesis of the antitumor compounds astins might be regulated. Since *A. tataricus* grows too large for laboratory studies, *A. thaliana* was chosen instead for co-cultivation experiments with *C. asteris* under sterile conditions on Petri dishes.

Co-cultivation assays with *A. thaliana* showed a root phenotype well known to be induced by IAA-producing microorganisms in plant-beneficial interactions [50,51,52]: shorter main root, increased lateral root growth, enhanced root biomass, and an auxin response detected in auxin-inducible promoter–reporter plants specifically in root tips and lateral root primordia.

A further question was whether *C. asteris* is capable of synthesizing IAA de novo and whether the IAA can also be secreted into the medium, and if so, by which pathway(s). In answering these questions, another piece would be added to the IAA biosynthesis puzzle in microorganisms and might contribute to resolving some of the loose ends. Genome analysis of genes possibly involved in IAA biosynthesis and feeding experiments with *C. asteris* using the IAA precursors and intermediates (Trp, TAM, IAN, IAM, IAD IPyA, indole) and the IAA biosynthesis inhibitors 2-mercaptobenzimidazole (MBI) [53] and yucasin DF (YDF) [54,55] will show us possible IAA biosynthetic routes.

In addition, the possible contribution of auxin uptake and efflux was analyzed for the co-cultivation between *C. asteris* and *A. thaliana* on plates containing IAA influx (2-naphthoxyacetic acid, NOA) [56] and efflux (N-1-naphtylphthalamic acid, NPA) inhibitors [57]. This could show if the root phenotype might be caused by fungal IAA secreted into the medium.

## 2. Results

### 2.1. The Endophyte C. asteris Induces a Root Phenotype on the Non-Host Plant A. thaliana that Is Reminiscent of High Auxin

**Figure 2:**
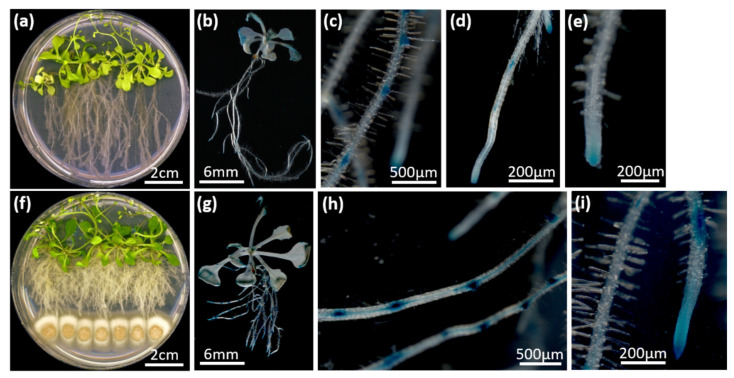
Phenotype of *A. thaliana* ecotype Columbia (Col-0) with *C. asteris*. *A. thaliana* and *C. asteris* were cultivated on ½ MS/MEAlow under long-day conditions. (**a**) Phenotype of *A. thaliana* Col-0 after 35 days of cultivation. (**b**–**e**) β-glucuronidase (GUS) staining of DR5::GUS lines of *A. thaliana* after 14 days of cultivation. (**f**) Phenotype of *A. thaliana* Col-0 co-cultivated with *C. asteris* after 35 days of cultivation. (**g**–**i**) GUS staining of roots of DR5::GUS lines of *A. thaliana* co-cultivated with *C. asteris* after 14 days of cultivation.

*C. asteris* induced a root phenotype with a massive lateral root and a reduced main root growth on the non-host plant *A. thaliana* that is reminiscent of auxin application (Figure 2a,f). The induction of the auxin response in root tips and lateral root primordia was also detected in a DR5::GUS line of *A. thaliana* (Figure 2b–e,g–i): Co-cultivated plants showed an increased amount of lateral root primordia that were additionally more intensely stained compared to controls.

Since the co-cultivation studies of *C. asteris* with *A. thaliana* showed an increased lateral root growth indicating a change in the plant IAA level, the possibility of an IAA biosynthesis by *C. asteris* was analyzed.

### 2.2. The Fungal Genome of C. asteris Reveals Several Putative Genes Involved in IAA Biosynthesis

Different candidate genes for IAA biosynthesis enzymes could be identified in the automated annotated genome sequence of *C. asteris* [58] (Table 1). Following the candidate genes, a TAM pathway would be possible, since all necessary genes were found in the genome of *C. asteris*. Three genes of the IAOx–IAN pathway were found, but two genes were missing for the IAOx dehydratase/hydrolase (IAOx into IAN) and the nitrile hydratase (IAN into IAM). Only one gene was found in the annotation for the IAM pathway, where the gene for the Trp monooxygenase in the first step (Trp into IAM) was missing. Genes for the conversion of Trp into IPyA were not found in the genome annotation of *C. asteris*, indicating that this pathway might not be used for IAA biosynthesis. In addition to the Trp-dependent pathways, there were also putative genes for the direct conversion of indole into IAA. However, since this is information from the genome and not transcriptome, it cannot be concluded that the genes detected would be indeed expressed.

### 2.3. C. asteris Secretes the Synthesized IAA into the Liquid Medium

**Figure 3:**
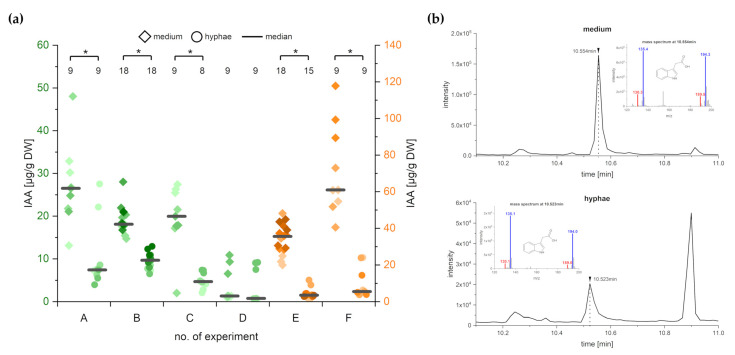
Amount of IAA in medium and hyphae of *C. asteris* in in vitro cultures. *C. asteris* was cultivated in MEAlow medium at 23 °C and 130 rpm in the dark for 10 days. (**a**) IAA levels in medium and hyphae of *C. asteris* in different experiments (A–F). Experiments A to D are shown with the left y-axis, experiments E and F are shown with the right y-axis. Data were analyzed using an unpaired t-test with Welch correction (test statistics can be found in Table S1). Significant differences between medium and hyphae are labeled with an asterisk. Sample size n is given in the upper part below the asterisks. (**b**) Extracted ion chromatogram of IAA (*m*/*z* 130) from medium and hyphae samples of *C. asteris*, including mass spectra (MS) at the specific retention times (red: MS data of IAA; blue: MS data of the internal standard D_5_-IAA).

Several candidate gene of *C. asteris* indicated a de novo biosynthesis of IAA, including different Trp-dependent pathways such as via TAM or IAOx-IAN but also a Trp-independent pathway with indole as the IAA precursor. Therefore, fungal in vitro cultures of *C. asteris* were analyzed for their IAA production after 10 days of cultivation (Figure 3). The specific mass spectrum of methylated IAA (Figure 3b), including the internal standard D_5_-IAA, was found in medium and hyphae of *C. asteris*. IAA was detected in all six experiments with higher IAA amounts in the medium compared to hyphae (Figure 3a, *p* < 0.05). *C. asteris* secreted most of the synthesized IAA into the medium (64% to 92%), since only 8% to 36% of the IAA was retained in the hyphae.

### 2.4. IAA Biosynthesis in C. asteris

**Figure 4:**
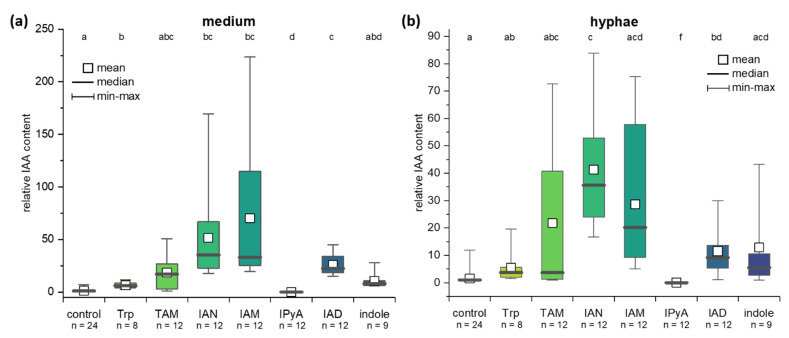
IAA levels in medium and hyphae of *C. asteris* after feeding with different IAA precursors. *C. asteris* was cultivated in MEAlow for seven days, before IAA precursors (Trp, TAM, IAN, IAM, IPyA, IAD, or indole) were added to the culture. IAA levels were detected after 10 days of cultivation (3 days with precursors). The IAA level in (**a**) medium and (**b**) hyphae of *C. asteris* is correlated to the respective control (without any IAA precursor), which is set to 1. Small letters above the box plots show the statistical significance; test statistics can be found in Table S2. Sample size n is given below the x-axis.

To elucidate the contribution of different possible pathways, *C. asteris* was incubated with different precursors and intermediates of IAA pathways (Figure 1) to evaluate their possible contribution to the biosynthesis. *C. asteris* was cultivated with 1 mM Trp, 1 mM TAM, 100 µM IAN, 100 µM IAM, 100 µM IPyA, 100 µM IAD, and 1 mM indole to induce the IAA biosynthesis (Figure 4). All precursors, except IPyA, increased the levels of IAA in *C. asteris*. IPyA is very unstable compared to other intermediates and was converted non-enzymatically to IAA (data not shown). In general, the direct precursors of IAA (IAN, IAM, and IAD) enhanced the biosynthesis very strongly in medium and hyphae (*p* < 0.05), whereas an intermediate earlier in the IAA biosynthesis, TAM, increased less strong and only in the hyphae. The early IAA biosynthesis precursors Trp and indole showed a small induction.

### 2.5. The IAA Biosynthesis Inhibitor MBI Reveals a Trp-Independent Biosynthesis Pathway in C. asteris

**Figure 5:**
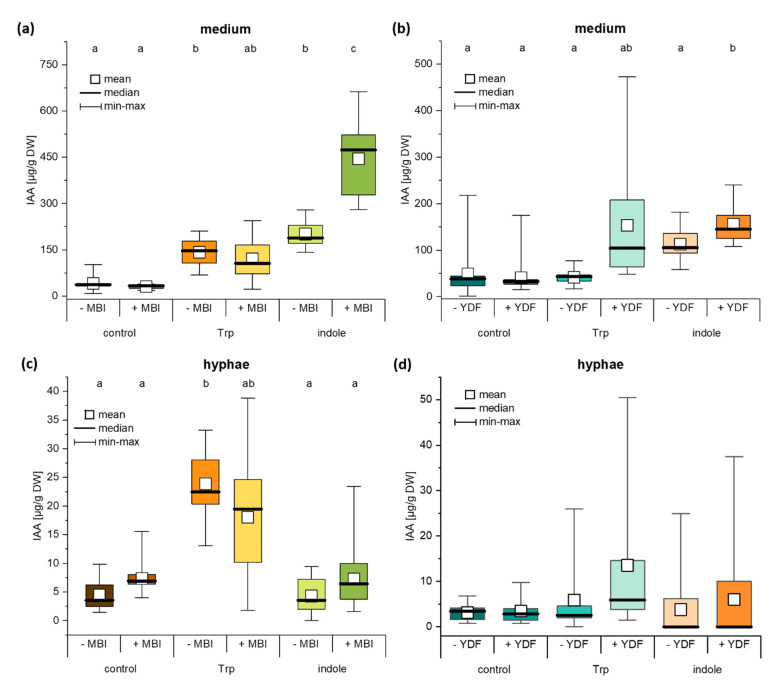
IAA content in medium and hyphae of *C. asteris* containing IAA precursors and IAA biosynthesis inhibitors. *C. asteris* was cultivated in MEAlow containing IAA biosynthesis inhibitors MBI or YDF. IAA precursors Trp and indole were added separately after 7 days of cultivation. IAA was detected after 10 days of cultivation. IAA content in (**a**) medium and (**c**) hyphae of *C. asteris* containing MBI as IAA biosynthesis inhibitor. IAA content in (**b**) medium and (**d**) hyphae of *C. asteris* containing YDF as IAA biosynthesis inhibitor. Small letters indicate statistically significant differences (Welch-ANOVA, post-hoc Games–Howell; see Table S3 for test statistics).

In addition to the Trp-dependent IAA biosynthesis, we were also interested in the possibility of a Trp-independent biosynthetic pathway. Here, two different IAA biosynthesis inhibitors MBI and YDF were used to disrupt the pathways and the two main IAA precursors Trp and indole were added to enhance possible effects (Figure 5).

The IAA biosynthesis inhibitor MBI increased the IAA level 4.2-fold in *C. asteris* cultures with indole (Figure 5a, *p* < 0.001). This was interpreted as the possibility to change to an alternative pathway from indole when the Trp-dependent one was blocked. The controls without any IAA precursor and with Trp did not change their IAA levels in the presence of MBI. Without any biosynthesis inhibitor, similar IAA levels were detected for Trp and indole as substrate in the cultures, and they were increased to the 4.4- to 5.5-fold compared to controls. These differences were only seen in the medium, not in hyphae (Figure 5c). Only Trp increased the IAA content in the hyphae, no matter whether MBI was present or not.

The addition of YDF, a second IAA biosynthesis inhibitor, did not change the IAA pattern as MBI did (Figure 5b). Controls without any IAA precursor and the cultures incubated with indole showed no significant differences between cultures with and without YDF. Only the cultures where Trp was added increased threefold their IAA levels, when YDF was present. Similar to MBI, the hyphae did not change their IAA levels when YDF was present (Figure 5d). This experiment showed evidence for a Trp-independent pathway to synthesize IAA from the precursor indole in *C. asteris*.

### 2.6. Auxin Synthesis by C. asteris and Uptake into the Host Plant Are Important for the Root Phenotype

**Figure 6:**
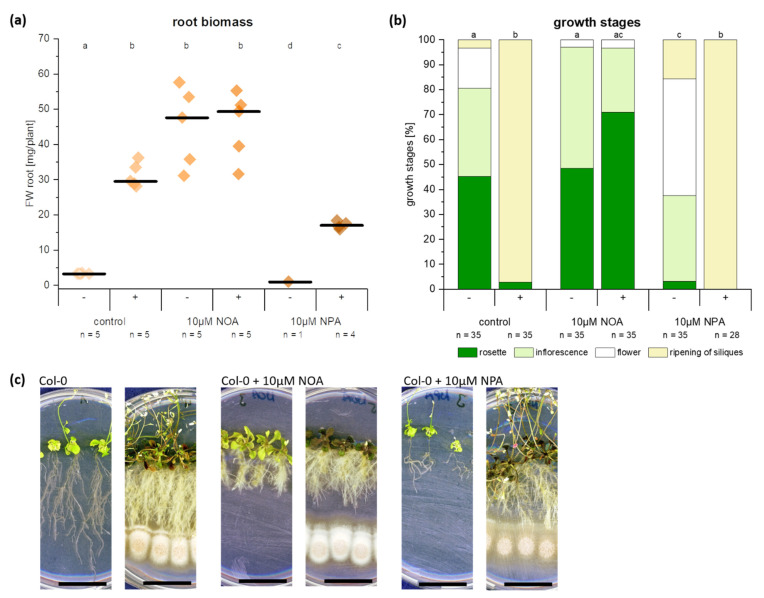
Co-cultivation of *A. thaliana* and *C. asteris* with IAA transport inhibitors 2-naphthoxyacetic acid (NOA) and N-1-naphtylphthalamic acid (NPA). *A. thaliana* and *C. asteris* were co-cultivated on ½ MS/MEAlow including either 10 µM NOA or 10 µM NPA under long day conditions for 35 days. (**a**) Root biomass and (**b**) growth stages of *A. thaliana* plants. Small letters indicate significant differences. –median. (**c**) Growth of *A. thaliana* and *C. asteris* on NOA or NPA containing medium. The bar indicates 2 cm. Statistical analyses can be found in Table S4.

The fungus *C. asteris* was co-cultivated with *A. thaliana* on medium containing two different IAA transport inhibitors: NOA and NPA (Figure 6). *A. thaliana* control plants developed a very strong phenotype in co-cultivation with *C. asteris*, including reduced main root length, massive lateral root growth, increased root and leaf biomass (Figure 2 and Figure S1). The addition of 10 µM NOA to the medium changed the phenotype of the *A. thaliana* plants: shorter main roots (Figure S1b), agravitropic root growth, and a higher root biomass (Figure 6a), resulting from the small, compact phenotype (Figure 6c) and a delayed plant development compared to the controls (Figure 6b). The co-cultivation with *C. asteris* changed the root phenotype of the NOA-treated plants only slightly: the root biomass (Figure 6a) and root length (Figure S1b) were similar; only the reduction of the root growth decreased slightly (Figure 6c). The plants co-cultivated with *C. asteris* developed similar to the plants without *C. asteris* on NOA (Figure 6b). NPA-treated plants had shorter roots (Figure S1b) and smaller rosettes (Figure S1a) compared to control plants; but they developed similar to controls with similar biomasses in leaves (Figure S1c) and roots (Figure 6a). Co-cultivation with *C. asteris* led in NPA-treated plants to longer roots (Figure S1b) and larger rosettes (Figure S1c) as seen already in control plants.

Increasing NOA and NPA concentrations in the medium (Figure S2) led to stronger effects: NOA-treated plants developed shorter roots and were delayed in plant development, which was not affected by *C. asteris*; NPA-treated plants showed still an enhanced lateral root growth and a faster plant development in the presence of *C. asteris*, but the main root growth was not reduced.

**Figure 7:**
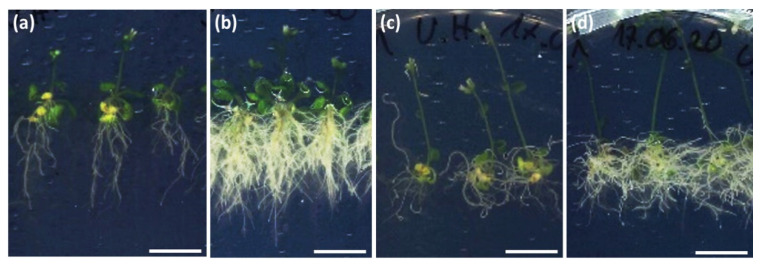
Root phenotype of ecotype Landsberg erecta (Ler-0) and auxin resistant 1 (*aux*1)-mutants of *A. thaliana* when incubated with *C. asteris* for 21 days. *A. thaliana* and *C. asteris* were co-cultivated on ½ MS/MEAlow under long day conditions. (**a**) Ecotype Ler-0 control, (**b**) ecotype Ler-0 co-cultivated with *C. asteris*. (**c**) *aux*1-mutant of *A. thaliana*, (**d**) *aux*1-mutant of *A. thaliana* co-cultivated with *C. asteris*. The bar indicates 1 cm.

*aux*1 mutants of *A. thaliana* (background: Landsberg erecta Ler-0) that lack the auxin resistant 1 (AUX1) influx carrier showed still an increased lateral roots growth (Figure 7c,d) in the presence of *C. asteris*. The agravitropic root growth, as seen in the *aux*1 controls, was still present, but it was not as strong as in the *aux*1 controls. The ecotype Ler-0 showed a similar root phenotype in the presence of *C. asteris* as the Columbia (Col-0) ecotype (Figure 2 and Figure 7a,b).

The mutant *tir*1, lacking the transport inhibitor response 1 (TIR1) receptor, showed a similar phenotype as already known from control plants with an increase in lateral roots and a reduction of main root growth, but no difference to Col-0 control roots (Figure S3). Since the TIR receptor family consists of several proteins, it is possible that others are responsible for IAA perception in the *tir1* mutant.

Summarizing these experiments, the IAA influx carrier inhibitor NOA reduced the effects caused by *C. asteris*, whereas NPA did not influence the phenotype of *A. thaliana* caused by *C. asteris*. The absence of the AUX1 influx carrier had a slight effect on the root phenotype, namely the agravitropic response was rescued by *C. asteris* to some extent.

## 3. Discussion

It was shown that the endophytic fungus *C. asteris* synthesized IAA via different pathways. In addition to Trp-dependent pathways, *C. asteris* uses also a Trp-independent pathway using indole as precursor to synthesize IAA. *C. asteris* secreted most of the IAA into the culture medium and retained only small amounts in its hyphae (Figure 3, Figure 4 and Figure 5). The root phenotype of *A. thaliana* co-cultivated with *C. asteris* was reminiscent of high auxin concentrations, and inoculated auxin-responsive promoter reporter lines (DR5-GUS) showed an increased staining in areas where lateral root primordia are formed compared to control roots (Figure 2). Similarly, it was reported for the interaction of *T. virens* and *A. thaliana* that in a DR5::GUS line, an increased activity in the root tip for auxin responses was found [1]. Unfortunately, the pictures did not reveal any changes in other parts of the roots, so it is not possible to compare these with the results found in the interaction of *C. asteris* and *A. thaliana* in this study (Figure 2).

Most of the IAA was secreted into the medium, indicating that fungal IAA most likely acts in the host plant and not in the fungal endophyte. In the natural environment inside the plant, secreted fungal IAA would influence the plant IAA homeostasis and thus plant AUX1 growth and development. IAA was synthesized using Trp-dependent and -independent pathways in *C. asteris* (for the different pathways, see Figure 1). The direct precursor of IAA in the Trp-dependent pathways—IAM, IAN and IAD—led to an increase in the IAA content, and this is corroborated by the existence of the candidate genes involved in the different pathways that were found in the genome of *C. asteris* (Table 1). Despite the fact that expression of the genes has not been determined so far, the increase of IAA production after incubation with the respective precursors indicate that these gene products could be involved in the auxin production.

The existence of the IPyA pathway could not be clearly verified, since IPyA did not increase the de novo synthesized IAA level in *C. asteris*. However, since IPyA was rapidly degraded in the fungal medium (data not shown), its possible role as an intermediate cannot completely be ruled out. In addition, candidate genes were not found in the genome, indicating that IAA might not be synthesized via the IPyA pathway. The IPyA pathway is widely spread in bacteria and fungi: *Azospirillum brasilense* [59,60], *Pseudomonas stutzeri* [61], and *Ustilago maydis* [24] are just a few examples. *Pseudomonas* species convert Trp directly into IAD without synthesizing IPyA, which is catalyzed by a Trp side chain monooxygenase [39,40,41,42]. Such a pathway cannot be excluded in *C. asteris*, but a candidate gene for a Trp monooxygenase in the genome was not found.

The IAM pathway would also be possible, but here again, a Trp monooxygenase would be needed. The first IAM pathway was described for *Pseudomonas savastanoi* [40], but it is found by now also in other plant-associated bacteria [62] and fungi. The fungal pathogens *Fusarium proliferatum* [27], *Colletotrichum fruticola* [63], and *C. gloeosporioides* f.sp. *aeschynomene* [64] synthesize IAA also over the IAM pathway. The latter fungus uses the IAM and IPyA pathway, preferring IAM as the main biosynthetic way [64]. The IAM pathway is usually used by pathogens to induce an uncontrolled plant cell growth [65]. However, different *Streptomyces* species indicate more than one IAA biosynthetic pathway, including the IAM pathway as the dominant one [66].

IAM is not only an intermediate in the IAM pathway, but it can also be an intermediate in the IAOx–IAN pathway. Candidate genes were found for the Trp conversion into IAOx in *C. asteris*, but no gene was found for the IAOx conversion into IAN. IAN can be converted either directly into IAA using nitrilases or via IAM into IAA, but the genome was lacking candidate genes for the nitrile hydratase (conversion of IAN into IAM) in *C. asteris*. Nitrilases are a large family in bacteria, fungi, plants, and animals [67]. IAN-specific nitrilases were found among others in *Pseudomonas* species [68,69] and *Alcaligenes faecalis* JM3 [70,71]. A nitrile hydratase could be isolated from the plant pathogen *Agrobacterium tumefaciens* and the leguminous *Rhizobium* spp. [72], which catalyzes the transformation of IAN into IAM.

The most possible IAA biosynthesis pathway in *C. asteris* might be the TAM pathway: here, candidate genes could be identified for each enzymatic reaction, and an induced biosynthesis with exogenous IAD could be shown (Figure 4). The TAM pathway is known in different bacteria and fungi. Several microorganisms are described to possess a Trp decarboxylase activity, which is responsible for the Trp decarboxylation of Trp to TAM (*Taphrina deformans* [73], *Metarhizium robertsii* [74]). Other microorganisms increase their IAA level when they were supplemented with exogenous TAM (*Azospirillium* [75], *Ustilago maydis* [76], *Leptosphaeria maculans* [6]). In addition to the TAM pathway, which might be the main pathway for IAA biosynthesis in *C. asteris*, there could exist also other pathways for IAA biosynthesis with partial enzymatic coverage, where the substrates for missing steps could be provided by the host plant.

In addition to the different Trp-dependent IAA biosynthesis pathways, there is a Trp-independent pathway. There is evidence that Trp-independent pathways exist in bacteria [60] and fungi [77], but it is not clear whether IAA is synthesized using indole or indole-3-glycerol phosphate (IGP) as a precursor. The IAA biosynthesis inhibitor MBI can inhibit the Trp-dependent biosynthesis of IAA [53,78]. *C. asteris* synthesizes high levels of IAA in the presence of indole (Figure 5), if the Trp-dependent biosynthesis was inhibited by MBI.

Different plant mutants suggest that IAA can be synthesized from indole and IGP. *Orp* mutants of maize, which show a defect in the last step of Trp biosynthesis [16,17], synthesized IAA from ^15^N_1_-anthranilate (indole precursor), but not from D_5_-Trp. The mutants accumulated up to 50 times more IAA than controls [79]. *trp*2 mutants of *A. thaliana* also synthesized IAA from ^15^N_1_-anthranilate and not from D_5_-Trp. *trp*2 and *trp*3 mutants accumulated very high levels of IAA (38-fold and 19-fold more than controls) [80]. *A. thaliana* plants with an antisense gene copy of IGP synthase as well as *trp*2-1 and *trp*3-1 mutants produced different amounts of IAA: whereas the *trp* mutants increased their IAA level, IGP antisense mutants decreased it [18]. Whether these results from plants can be transferred to bacteria and fungi has to be shown by further research, but there is a strong evidence for the existence of a Trp-independent IAA biosynthesis starting with indole and/or IGP also in these organisms.

The second IAA biosynthesis inhibitor YDF used in our study is a more stable derivative of yucasin and blocks the conversion of Trp into IPyA by the YUCCA enzymes in plants [54,55]. The application of YDF to *C. asteris* changed the IAA levels in Trp-treated cultures but not dramatically. The IPyA pathway is only one out of many different IAA biosynthesis pathways, which could compensate the possible reduction of IAA. Furthermore, possible genes involved in the IPyA pathway were not found in the genome, which does not exclude them since it is only an automatic annotation. The cultures supplemented with indole showed an increased IAA level indicating alternative IAA biosynthesis pathways in *C. asteris* beside over IPyA. In consequence, the IAA level increased only very slightly (not significant) in these cultures in the presence of YDF (Figure 5).

In the co-cultivation experiment with *A. thaliana* and *C. asteris* on medium containing the IAA transport inhibitors NOA or NPA, the IAA efflux carrier inhibitor NPA did not influence the root phenotype remodeling associated with *C. asteris*. Only the IAA influx carrier inhibitor NOA inhibited the alterations in the root architecture of *A. thaliana* in most instances. The roots developed, compared to the non-co-cultivated plants on NOA, only slightly elongated lateral roots without an increase in root biomass. IAA is transported into the cell passively by diffusion and actively by AUX1/LAX transporters [81]. These transporters are blocked by NOA [56], reducing the intracellular IAA level. Diffusion of IAA still takes place and explains why NOA-treated plants slightly increased their root length in the presence of *C. asteris*. NPA is targeting the IAA efflux carriers ABCB1 and ABCB19 [82,83,84], which blocks the polar and thus basipetal auxin transport [10]. There is disagreement regarding whether NPA directly inhibits the PIN efflux transporters [57]. *pin*1 loss-of-function mutants of *A. thaliana* show the same phenotype as NPA-treated plants [85,86,87]. NPA-treated plants showed the typical phenotype, described in the literature [88]: agravitropic root growth with reduced lateral roots. The influx carrier proteins are not affected by NPA [89], so IAA from the fungus can still be transported into the cell and influence the IAA homeostasis of the plant.

*A. thaliana* plants without any NOA developed a root phenotype (Figure 2 and Figure 6), which is known to be induced by other plant-beneficial microorganisms. Treating plants with IAA transport inhibitor reduces the effect of microorganisms on root architecture [90,91,92,93]. For example, the plant-growth promoting fungus *Aspergillus ustus* induced lateral root growth and thus root biomass [94]. The bacterium *Bacillus megaterium* STM196 reduced primary root growth, but it induced lateral root growth and root biomass [51]. Several microorganisms are known to induce these changes in root architecture (reviewed in [95]). The induction of lateral root growth was lost in *aux*-mutants of *A. thaliana* when inoculated with different microorganisms [91,96]. The increased root system, induced by plant-beneficial microorganisms, can improve nutrition uptake, which leads to a better tolerance against different environmental stresses [97,98,99].

Further experiments should investigate the potential contribution of the different candidate genes involved in IAA biosynthesis that were identified. Additional feeding experiments with heavy-isotope labeled Trp and identification of the intermediates will also improve our knowledge about which Trp-dependent pathway is preferred by *C. asteris*.

## 4. Materials and Methods

### 4.1. Plant and Fungal Material

All *A. thaliana* ecotypes (Col-0 and Ler-0) and mutants (*aux*1 and *tir*1) used in this study were obtained from Nottingham Arabidopsis Stock Centre (NASC, Loughborough, UK). *C. asteris* was derived from our own culture (deposited under the accession number DSM 100826 at DSMZ, Braunschweig, Germany).

### 4.2. Co-Cultivation of A. thaliana with C. asteris

*A. thaliana* plants of the ecotypes Col-0, Ler-0, and auxin mutants *aux*1 and *tir*1 were co-cultivated with *C. asteris* on Petri dishes, sealed with Parafilm (Bemis, Neenah, WI, USA). *A. thaliana* seeds were sterilized with 70% ethanol containing 0.1% Triton-X 100 for 1 min, followed by 1.2% sodium hypochlorite containing 0.1% Triton-X 100 and three to four times washing with autoclaved water. Seven sterilized seeds were sown in the upper part of Petri dishes with ½ MS/MEAlow (1.1 g/L MS incl. vitamins, 5 g/L malt extract, 5 g/L D-glucose, 0.5 g/L peptone, 0.5 mL/L Hutner’s trace elements [100]; pH 6.1) and cultivated under long day conditions (16 h with 23°C with light intensities between 90 and 120 µmol s^−1^ m^−2^; 8 h with 18 °C in the dark). After one week, *C. asteris* conidia were applied to the lower part of the Petri dish. Conidia were harvested from a one-week-old *C. asteris* culture in MEAlow by filtering (gauze, 20 µm mesh size) and centrifugation (5000 rcf, 4 °C, 10 min). The pellet with conidia was dissolved in MEAlow and used for inoculation. Each plant was inoculated with 10^5^ conidia in a distance of 4.5 cm to the seeds (seven spots per Petri dish). The phenotype of *A. thaliana* plants was recorded weekly (root length, growth stage). The plants were harvested after 35 days of cultivation for fresh and dry weight determination. For treatment with auxin transport inhibitors, *A. thaliana* seeds were sterilized and sown as above. After one week, *A. thaliana* seedlings were transferred to Petri dishes containing ½ MS/MEAlow including NOA (10 µM and 30 µM; dissolved in water and filter sterilized) and NPA (10 µM and 20 µM; dissolved in DMSO), and co-cultivated with *C. asteris* conidia as described above.

### 4.3. GUS Staining of A. thaliana DR5::GUS

Harvested plants of *A. thaliana* DR5::GUS were incubated in 5-bromo-4-chloro-1*H*-indol-3-yl β-D-glucopyranosiduronic acid (X-Gluc) incubation buffer (0.1 M phosphate buffer, 10 mM Na2EDTA, 0.5 M K_3_[Fe(CN_6_)], 0.5 M K_4_[Fe(CN_6_)], 0.5% Triton X-100; filter sterilized and 1.2 µM X-Gluc added) for 2 h at 37 °C in the dark. Reaction was stopped by transferring plants into 0.1 M phosphate buffer. Green tissues were destained in ethanol (ascending order of 30%–50%–70%–90%–100%). The plants were screened with an Axio Zoom.V16 (Carl Zeiss Microscopy, Jena, Germany).

### 4.4. Incubation of C. asteris with IAA Precursors and/or Biosynthesis Inhibitors

*C. asteris* was cultivated in liquid MEAlow on a shaking incubator (23 °C, 130 rpm) for 10 days. After centrifugation (5000 rcf, 10 °C, 10 min) to separate medium and hyphae, hyphae were freeze-dried and dry weight was determined. IAA was extracted and analyzed from medium and freeze-dried hyphae.

To determine an IAA biosynthesis pathway in *C. asteris*, liquid cultures of *C. asteris* were fed with different IAA precursors (1 mM Trp dissolved in water, sterilized by autocaving, and 1 mM indole dissolved in methanol) and intermediates (1 mM TAM; 100 µM of IAN/ IAM/IAD/IPyA; all dissolved in methanol) after seven days of cultivation. The cultures were harvested (see above) after three additional days for IAA extraction and analysis (see Section 4.4).

In a third experiment, *C. asteris* was cultivated in liquid MEAlow including the IAA biosynthesis inhibitors MBI (60 µM; dissolved in isopropanol) or YDF (100 µM; dissolved in isopropanol). IAA precursors Trp or indole (each 1 mM) were added after seven days. After an additional three days, the fungal culture was harvested (see above) to extract and analyze the IAA content.

### 4.5. Extraction and Detection of IAA

For IAA extraction, each medium sample was divided into three technical replicates, which was set up with 1 µg internal standard D_5_-IAA (Euriso-top SAS, Saint-Aubin, France). Then, the medium was shaken for 2 h in the dark at 4 to 8 °C to guarantee a uniformly distribution of the internal standard. After this, the pH value was set to three, and the medium was extracted twice with the same volume of ethyl acetate (HPLC grade, Carl Roth, Karlsruhe, Germany) (vortexing for 10 s, centrifugation for 5 min, 4 °C, 5000 rcf). The organic phases were collected and evaporated at a Multivapor P-12 (Büchi Labortechnik GmbH, Essen, Germany). The dried sediments were resuspended twice in 500 µL methanol (HPLC grade, Carl Roth, Karlsruhe, Germany) and again evaporated under gaseous nitrogen. In the next step, the 200 µL of methanol resolved IAA was methylated with the same volume of (trimethylsilyl)diazomethane (1:100 dilution in diethylether) at room temperature for 30 min, before the methanol was evaporated under gaseous nitrogen. The IAA sample was finally resuspended in 50 µL of ethyl acetate and measured by gas chromatography-mass spectrometry (GC-MS).

Freeze-dried hyphae samples were ground and mixed with 80% methanol, including 5% acetic acid. The samples were divided into three technical replicates, and 1 µg internal standard D_5_-IAA was added to each replicate. After shaking for 2 h in the dark at 4 to 8 °C, the samples were centrifuged to remove the pellet (10,000 rcf, 4 °C, 10 min). The methanol of the supernatant was evaporated under gaseous nitrogen until the water phase was left. The pH of the water phase was set to three and extracted twice with the same volume of ethyl acetate. The combined organic phases of ethyl acetate were evaporated under gaseous nitrogen before the methylation of IAA was performed as described above. The sample was treated as described for medium, and the IAA contents were measured by GC-MS.

Samples were separated by GC 3900 on an Agilent HP-5 column (30 m/Ø 0.250 mm/0.25 µm film) with 1 mL/min helium (10 psi). Sample (1 µL) was injected splitless at 250 °C and separated by the following method: 2 min at 70 °C, then a temperature increase about 20 °C/min until 280 °C, and held until 17.5 min. The separated sample was analyzed with an ion trap (Saturn 2100 MS; Varian, Palo Alto, CA, USA) and ionized by electron ionization (70 eV) at 200 °C.

The IAA content of each sample was calculated according to the isotope dilution equation [101] with the corresponding *m*/*z* (methylated IAA: *m*/*z* 130; methylated D_5_-IAA: *m*/*z* 135). The background of the IAA precursors and intermediates at m/z 130 were subtracted to get only the de novo synthesized IAA levels of *C. asteris*. To compare the different experiments with the different IAA precursors/intermediates, the IAA levels in each experiment were related to the respective control without any supplementation.

### 4.6. Data Analysis

Statistical analyses were carried out using OriginPro 2021 version 9.8.0.200 (OriginLab Corporation, Northampton, MA, USA).

Data of the interaction study between *A. thaliana* and *C. asteris* on NOA- or NPA-containing medium were analyzed with different statistical analyses: (a) A Welch-ANOVA (due to an unequal sample size and dissimilar variances) was used for biomarkers with unequal samples size and dissimilar variances, with IAA transport inhibitor included as a six-level between-subjects factor (control, NOA, NPA without and with *C. asteris*). Post-hoc tests were performed using a Games–Howell test. (b) A Kruskal–Wallis test was used for biomarkers with equal sample size but unsimilar variances or for growth stages, with IAA transport inhibitor included as a three-level (control, NOA, NPA) and *C. asteris* included as two-level (without or with *C. asteris*) between-subjects factor. Post-hoc tests were performed using Dunn’s test. Effect sizes were calculated [102,103].

For determination of biosynthetic pathways, data of the controls (without any precursor or intermediate) were analyzed using an unpaired t-test on the medians of the three technical replicates, assuming unequal variances (Welch correction).

IAA levels of *C. asteris* cultures, which were supplemented with different IAA precursors/intermediates and/or IAA biosynthesis inhibitors, were statistically analyzed by performing a Welch-ANOVA on the medians of the three technical replicates, with IAA precursors as an eight-level (control, Trp, TAM, IAN, IAM, IPyA, IAD, indole) or IAA biosynthesis inhibitor as a six level (control, MBI, and YDF without and with *C. asteris*) between-subjects factor. A post-hoc test was performed (Games–Howell test) and effect sizes were calculated [102,103]. The respective test used is given in the results and/or figure legends.

## 5. Conclusions

This study revealed that the endophytic fungus *C. asteris* produces the plant hormone IAA via different biosynthetic pathways: Trp-dependent and Trp-independent. The most likely Trp-dependent way is the TAM pathway, which does not exclude other pathways such as via IPyA or IAM. The Trp-independent pathway starts with indole as the substrate. By synthesizing and secreting IAA, *C. asteris* influences the root growth of *A. thaliana* that results in more lateral but shorter main roots. The phenotype was reduced after the addition of an auxin transport inhibitor for influx but not efflux. Further experiments should lead to the identification of responsible genes in the endophyte for the biosynthesis and secretion of IAA. This study needs to be investigated further in a future study to see how the growth of the roots behave and change through different stages of the plant growth ending to the greenhouse stage.

## Figures and Tables

**Table 1:** Candidate genes of *C. asteris* involved in IAA biosynthesis. Shown are the candidate genes in *C. asteris* to the corresponding enzyme reaction, but certainly this is no evidence that they are expressed in *C. asteris*. The putative IAA biosynthesis pathways in *C. asteris* are highlighted in orange in Figure 1.

Reaction	Enzyme	Candidate Genes (Gene ID)
Trp → TAM	Trp decarboxylase	1234
TAM → IAOx	flavin monooxygenase enzyme *	17, 2090, 4995, 5792, 7832 2451, 3001, 6023, 9811
TAM → IAD	monoamine oxidase	3466, 3467, 8359
IAD → IAA	IAD dehydrogenase	10233, 6228, 7132, 8151, 9123
Trp → IAOx	cytochrome P450	17, 2090, 4995, 5792, 7832
IAOx → IAN	IAOx dehydratase/hydrolase	-
IAN → IAA	nitrilase	1223, 5809
IAN → IAM	nitrile hydratase	-
Trp → IAM	Trp monooxygenase	-
IAM → IAA	IAM hydrolase	1862, 6419
Trp → IPyA	Trp aminotransferase	-
IPyA → IAD	IPyA decarboxylase	-
indole → IAA	-	2451, 3001, 6023, 9811

* This reaction involves two different enzymes (TAM → N-hydroxy-TAM → IAOx).

## Data Availability

All data supporting this study are available in this paper and in its supplementary data published online.

## Supplementary Materials

The following are available online at https://www.mdpi.com/1422-0067/22/5/2651/s1, Figure S1: Co-cultivation of *A. thaliana* and *C. asteris* on IAA transport inhibitor containing medium, Figure S2: Co-cultivation of *A. thaliana* and *C. asteris* in the presence of IAA transport inhibitors, Figure S3: Growth of *tir*1 mutant of *A. thaliana* with *C. asteris* after 35 days, Table S1: Test statistics on IAA levels in medium and hyphae of *C. asteris,* using an unpaired *t*-test. Table S2: Test statistics on IAA levels in medium and hyphae of *C. asteris* in presence of IAA precursors, Table S3: Test statistics on IAA levels in medium and hyphae of *C. asteris* in the presence of MBI or YDF, Table S4: Test statistics on co-cultivation of *A. thaliana* and *C. asteris* on 10 µM NOA or 10 µM NPA, Table S5: Test statistics on co-cultivation of *A. thaliana* and *C. asteris* on 30 µM NOA or 20 µM NPA. Table S6: Raw data.
