# Supplementary material for: Indole-3-Acetic Acid Is Synthesized by the Endophyte Cyanodermella asteris via a Tryptophan-Dependent and -Independent Way and Mediates the Interaction with a Non-Host Plant
Source: Int J Mol Sci. 2021 Mar 6;22(5):2651. doi: 10.3390/ijms22052651 (PMC7961953; doi:10.3390/ijms22052651)
Supplement: Supplementary file 1 [file ijms-22-02651-s001.zip › Supplementary files/Supplementary figures S1-S3 MDPI.docx]

Supplementary figures


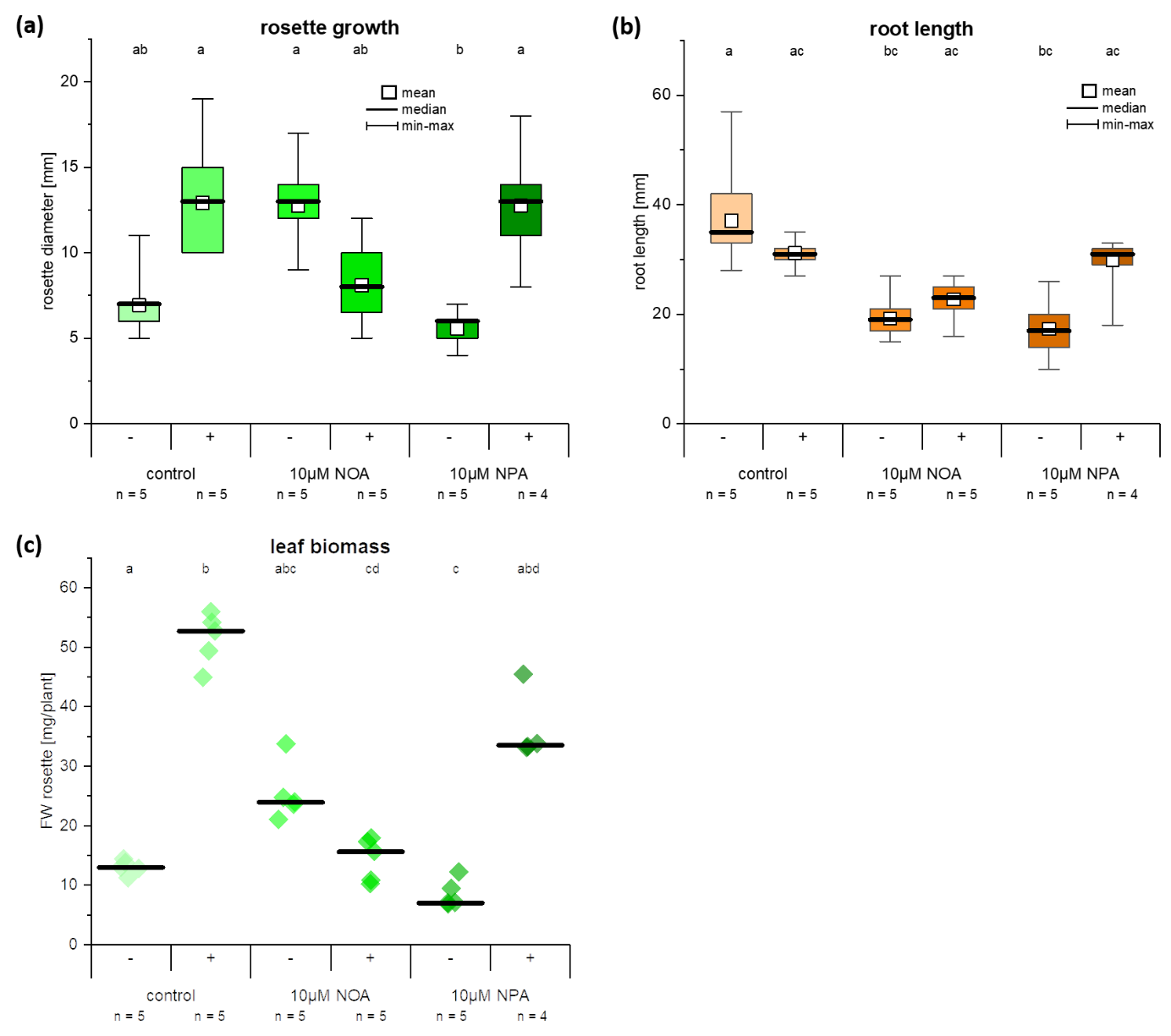


**Figure S1.** Co-cultivation of *A. thaliana* and *C. asteris* on IAA transport inhibitor containing medium. *A. thaliana* and *C. asteris* were co-cultivated on ½ MS/ MEAlow including 10µM NOA or 10µM NPA under long day conditions for 35 days. (a) Rosette diameter, (b) main root length and (c) leaf biomass of A. thaliana plants. Statistical analyses can be found in Table S4.


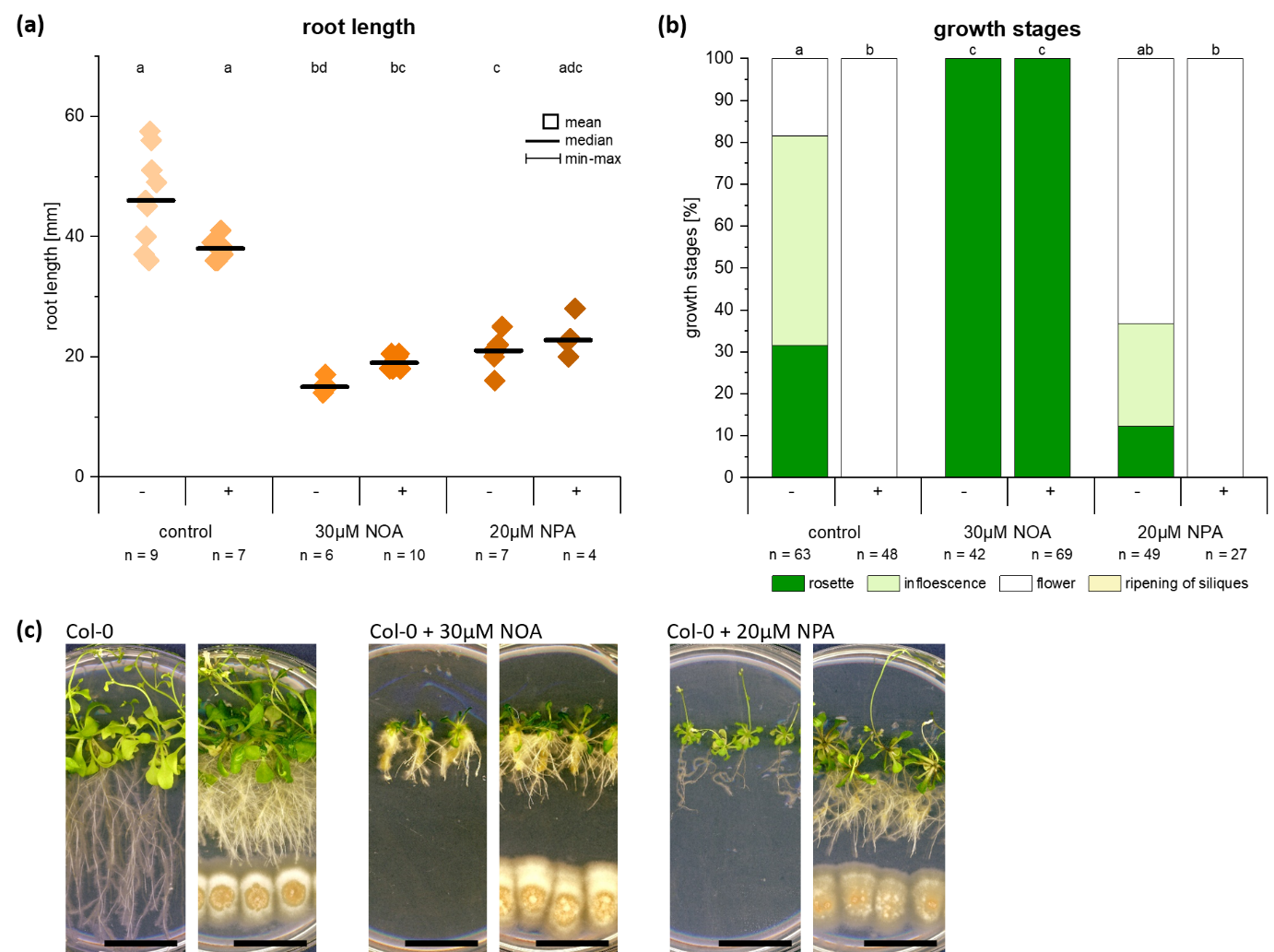


**Figure S2.** Co-cultivation of *A. thaliana* and *C. asteris* in the presence of IAA transport inhibitors. *A. thaliana* and *C. asteris* were co-cultivated on ½ MS/MEAlow medium including NOA (30µM) or NPA (20µM) under long day conditions for 35 days. (a) Main root length, (b) growth stages, and (c) growth of A. thaliana. Statistical analyses can be found in Table S5.


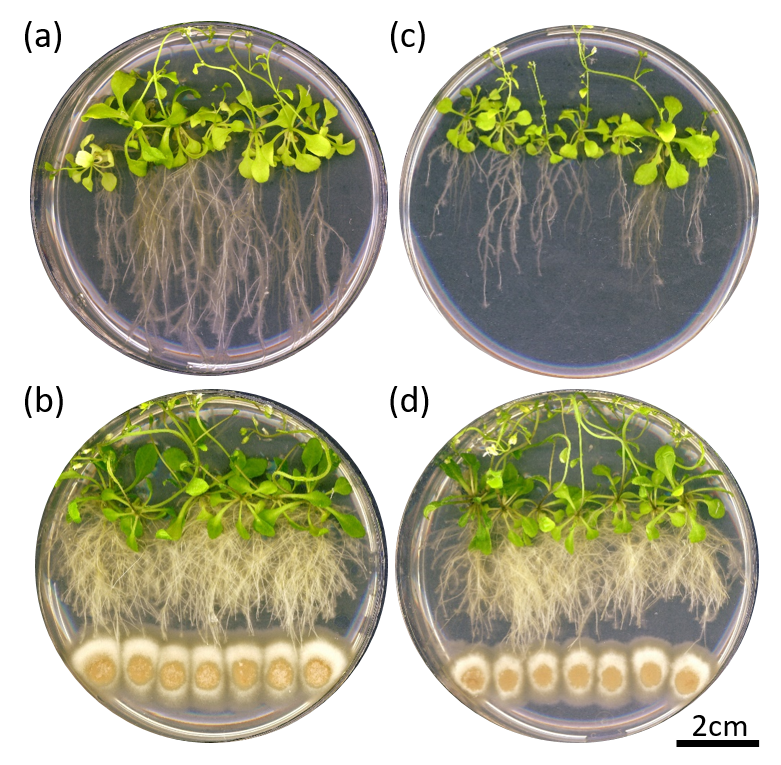


**Figure S3.** Growth of *tir*1 mutant of *A. thaliana* with *C. asteris* after 35 days. *A. thaliana* and *C. asteris* were co-cultivated on ½ MS/MEAlow under long day conditions. (a) Growth of *A. thaliana* Col-0 ecotype, (b) growth of *A. thaliana* *tir*1 mutant, (c) growth of *A. thaliana* Col-0 with *C. asteris*, (d) growth of *A.thaliana* *tir*1 mutant with *C. asteris*.
