# Supplementary material for: Indole-3-Acetic Acid Is Synthesized by the Endophyte Cyanodermella asteris via a Tryptophan-Dependent and -Independent Way and Mediates the Interaction with a Non-Host Plant
Source: Int J Mol Sci. 2021 Mar 6;22(5):2651. doi: 10.3390/ijms22052651 (PMC7961953; doi:10.3390/ijms22052651)
Supplement: Supplementary file 1 [file ijms-22-02651-s001.zip › Supplementary files/Supplementary Tables S1-S5_MDPI.docx]

Supplementary tables

**Table S1.** Test statistics on IAA levels in medium and hyphae of *C. asteris,* using an unpaired t-test. dF … degrees of freedom, t … t-statistic, p … probability, g_s_ … effect size Hedge’s g_s_ (0.2-0.5 small effect, 0.5-0.7 moderate effect, >0.8 large effect).

| **experiment** | **dF** | **t** | **p** | **g_s_** |
| --- | --- | --- | --- | --- |
| A | 16 | 3.91 | <0.001 | 1.76 |
| B | 26.94 | 11.76 | <0.001 | 3.83 |
| C | 9.23 | 5.50 | <0.001 | 2.40 |
| D | 16 | 0.25 | 0.81 |  |
| E | 21.70 | 15.07 | <0.001 | 4.77 |
| F | 9.77 | 6.97 | <0.001 | 3.13 |

Effect size g_s_ was calculated using

[1].

**Table S2.** Test statistics on IAA levels in medium and hyphae of *C. asteris* in presence of IAA precursors. Performed with a Welch-ANOVA, including IAA precursor as an eight-level between-subject factor. Post-hoc test was performed using a Games-Howell test. dF … degrees of freedom, F … F-statistic, p … probability, η_p_^2^ … effect size (0.01-0.06 small effect, 0.06-0.14 moderate effect, 0.14-1 large effect).

|  | **dF_1_** | **dF_2_** | **F** | **p** | **η_p_^2^** |  |
| --- | --- | --- | --- | --- | --- | --- |
| **medium** | 7 | 30.31 | 27.00 | <0.001 | 0.86 |  |
|  | control  Trp  TAM  IAN | a  b  abc  bc | IAM  IPyA  IAD  indole | bc  d  c  abd |  |  |
| **hyphae** | 7 | 29.54 | 12.97 | <0.001 | 0.76 |  |
|  | control  Trp  TAM  IAN | a  ab  abc  c | IAM  IPyA  IAD  indole | acd  f  bd  acd |  |  |

Effect size η_p_^2^ was calculated using

[1].

**Table S3.** Test statistics on IAA levels in medium and hyphae of *C. asteris* in the presence of MBI or YDF. Performed was a Welch-ANOVA with IAA precursor as a six level (control, Trp, indole without and with IAA biosynthesis inhibitor) between-subjects factor. Post-hoc tests were performed using a Games-Howell test. dF … degrees of freedom, F … F-statistic, p … probability, η_p_^2^ … effect size (0.01-0.06 small effect, 0.06-0.14 moderate effect, 0.14-1 large effect).

|  | | **test statistics** | | | | |
| --- | --- | --- | --- | --- | --- | --- |
| **MBI** | **medium** | dF_1_=5 | dF_2_=13.08 | F=139.92 | p<0.001 | η_p_^2^=0.98 |
|  |  | control –  control +  Trp – | a  a  b | Trp +  indole –  indole + | ab  b  c |  |
|  | **hyphae** | dF_1_=5 | dF_2_=12.66 | F=8.65 | p<0.001 | η_p_^2^=0.77 |
|  |  | control –  control +  Trp – | a  a  b | Trp +  indole –  indole + | ab  a  a |  |
| **YDF** | **medium** | dF_1_=5 | dF_2_=13.64 | F=32.24 | p<0.001 | η_p_^2^=0.92 |
|  |  | control –  control +  Trp – | a  a  a | Trp +  indole –  indole + | ab  a  b |  |
|  | **hyphae** | dF_1_=5 | dF_2_=13.28 | F=0.92 | p>0.5 | η_p_^2^=0.26 |
|  |  | control –  control +  Trp – | a  a  a | Trp +  indole –  indole + | a  a  a |  |

Effect size η_p_^2^ was calculated using

[1].

**Table S4**. Test statistics on co-cultivation of *A. thaliana* and *C. asteris* on 10µM NOA or 10µM NPA. dF … degrees of freedom, F … F-statistic, p … probability, η_p_^2^ and η^2^ … effect sizes (0.01-0.06 small effect, 0.06-0.14 moderate effect, 0.14-1 large effect).

| **variable** | **test statistics** | | | | | | | | |
| --- | --- | --- | --- | --- | --- | --- | --- | --- | --- |
| **root biomass** | Welch-ANOVA, with IAA transport inhibitor as a six level (control, NOA, NPA without or with *C. asteris*) between-subjects factor | | | | | | | | |
|  | dF_1_=5 | dF_2_=8.75 | F=238.91 | | p<0.001 | | | η_p_^2^=0.99 | |
|  | post-hoc test: Games-Howell (α=0.05) | | control –  control +  NOA – | a  b  b | NOA +  NPA –  NPA + | b  d  c | | | |
| **leaf biomass** | Kruskal-Wallis test, with IAA transport inhibitor as a six level (control, NOA, NPA without or with *C. asteris*) between-subjects factor | | | | | | | | |
|  |  | dF=5 | Χ^2^=25.46 | | p<0.001 | | | η^2^=0.89 | |
|  | post-hoc test: Dunn’s (α=0.05) | | control –  control +  NOA – | a  b  abc | NOA +  NPA –  NPA + | cd  c  abd | | | |
| **root length** | Kruskal-Wallis test, with IAA transport inhibitor as a six level (control, NOA, NPA without or with *C. asteris*) between-subjects factor | | | | | | | | |
|  |  | dF=5 | Χ^2^=25.82 | | p<0.001 | | | η^2^=0.91 | |
|  | post-hoc test: Dunn’s (α=0.05) | | control –  control +  NOA – | a  ac  bc | NOA +  NPA –  NPA + | ac  bc  ac | | | |
| **rosette diameter** | Kruskal-Wallis test, with IAA transport inhibitor as a six level (control, NOA, NPA without or with *C. asteris*) between-subjects factor | | | | | | | | |
|  |  | dF=5 | Χ^2^=23.00 | | p<0.001 | | | η^2^=0.78 | |
|  | post-hoc test: Dunn’s (α=0.05) | | control –  control +  NOA – | ab  a  a | NOA +  NPA –  NPA + | ab  b  a | | |  |
| **growth stages** | Kruskal-Wallis test, with IAA transport inhibitor as a six level (control, NOA, NPA without or with *C. asteris*) between-subjects factor | | | | | | | | |
|  |  | dF=5 | Χ^2^=99.94 | | p<0.001 | | | η^2^=0.48 | |
|  | post-hoc test: Dunn’s (α=0.05) | | control –  control +  NOA – | a  b  a | NOA +  NPA –  NPA + | | ac  c  b |  | |

Effect size η_p_^2^ was calculated using

[1] and effect size η^2^ was calculated using

[2].

**Table S5.** Test statistics on co-cultivation of *A. thaliana* and *C. asteris* on 30µM NOA or 20µM NPA. dF … degrees of freedom, F … F-statistic, p … probability, η_p_^2^ and η^2^ … effect sizes (0.01-0.06 small effect, 0.06-0.14 moderate effect, 0.14-1 large effect).

| **variable** | **test statistics** | | | | | | |
| --- | --- | --- | --- | --- | --- | --- | --- |
| **root length** | Welch-ANOVA, with IAA transport inhibitor as a six level (control, NOA, NPA without or with *C. asteris*) between-subjects factor | | | | | | |
|  | dF_1_=5 | dF_2_=14.21 | F=158.26 | p<0.001 | | η_p_^2^=0.98 | |
|  | post-hoc test: Games-Howell (α=0.05) | | control –  control +  NOA – | a  a  bd | NOA +  NPA –  NPA + | | bc  c  adc |
| **growth stages** | Kruskal-Wallis test, with IAA transport inhibitor as a six level (control, NOA, NPA without or with *C. asteris*) between-subjects factor | | | | | | |
|  |  | dF=5 | Χ^2^=261.60 | p<0.001 | | η^2^=0.88 | |
|  | post-hoc test: Dunn’s (α=0.05) | | control –  control +  NOA – | a  b  c | NOA +  NPA –  NPA + | | c  ab  b |

Effect size η_p_^2^ was calculated using

[1] and effect size η^2^ was calculated using

[2].

References

1. Lakens, D. Calculating and Reporting Effect Sizes to Facilitate Cumulative Science: A Practical Primer for T-tests and ANOVAs. *Frontiers in Psychology* **2013**, 4, doi:10.3389/fpsyg.2013.00863.

2. Lenhard, W.; Lenhard, A. Calculation of Effect Sizes. *Dettelbach (Germany): Psychometrica* 2016. Available online: <https://www.psychometrica.de/effect_size.html> (accessed 12/01/2021), doi: 10.13140/RG.2.2.17823.92329.
